# Urinary Angiotensinogen-Melatonin Ratio in Gestational Diabetes and Preeclampsia

**DOI:** 10.3389/fmolb.2022.800638

**Published:** 2022-03-02

**Authors:** Gabriela Ribeiro Valias, Patricia Rodrigues Lourenço Gomes, Fernanda G. Amaral, Saif Alnuaimi, Daniela Monteiro, Siobhán O'Sullivan, Renato Zangaro, José Cipolla-Neto, Juan Acuna, Ovidiu Constantin Baltatu, Luciana Aparecida Campos

**Affiliations:** ^1^ Center of Innovation, Technology and Education (CITE) at Anhembi Morumbi University–Anima Institute, Sao Jose dos Campos Technology Park, Sao Jose dos Campos, Brazil; ^2^ Department of Physiology and Biophysics, Institute of Biomedical Sciences, University of São Paulo, São Paulo, Brazil; ^3^ Department of Physiology, Federal University of São Paulo, São Paulo, Brazil; ^4^ Department of Public Health and Epidemiology, Khalifa University, Abu Dhabi, United Arab Emirates; ^5^ Department of Molecular Biology and Genetics, Khalifa University, Abu Dhabi, United Arab Emirates

**Keywords:** angiotensinogen, melatonin, preeclampsia, gestational diabetes, biomarker

## Abstract

**Background:** A large research portfolio indicates that an activated renal renin-angiotensin system or a deficit on melatonin is associated with several cardiovascular pathologies. In this observational clinical study, we hypothesized that alterations in urinary melatonin or angiotensinogen levels may be altered in two common conditions, preeclampsia and gestational diabetes. Our study’s primary objective was to assess melatonin and angiotensinogen as novel disease biomarkers detectable and quantifiable in the urine of pregnant women with or without pregnancy complications.

**Methods:** This was a concurrent cohort study of pregnant women with selected obstetric pathologies (gestational diabetes, preeclampsia, hypertension and obesity with hypertension). A group of healthy controls was also included. Urinary 6-sulfatoxymelatonin and angiotensinogen were measured by sensitive and specific ELISAs in first morning void urine samples. The patients were included in the cohort consecutively, and the diagnosis was blinded at the level of urine collection. Urinary 6-sulfatoxymelatonin and angiotensinogen levels were investigated in the patients included in the cohort.

**Results:** Urinary levels of angiotensinogen were significantly higher in the gestational diabetes [angiotensinogen/creatinine ratio median (25th, 75th): 0.11 (0.07, 0.18)] and preeclampsia [0.08 (0.06, 0.18)] groups than in those with healthy pregnancy [0.05(0.04, 0.06]; 6-sulfatoxymelatonin levels were significantly lower in the gestational diabetes [ug/h: median (25th, 75th): 0.12(0.08, 0.17)] and preeclampsia [0.12 (0.09, 0.15)] groups than in those with healthy pregnancy [0.20 (0.15, 0.27]. Neither morning void protein/creatinine ratio nor 24-h urine protein estimate were significantly different between the study groups.

**Conclusion:** These results suggest that urinary angiotensinogen levels may indicate an intrarenal RAS activation while melatonin production appears to be defective in gestational diabetes or hypertension. An angiotensinogen/melatonin ratio is suggested as an early biomarker for identification of gestational diabetes or hypertension. This report provides a basis for the potential use of melatonin for the treatment of preeclampsia. A prospective study in a larger number of patients to determine the operative characteristics of these markers as potential diagnostic tests is justified.

## Introduction

Preeclampsia and gestational diabetes mellitus are two common complications of pregnancy including cerebral and cardiac incidents, multiorgan failure such as end-stage renal disease (ESRD) and are the major causes of perinatal morbidity and mortality where early identification of those at risk is highly beneficial (Vadalà et al., 2017; Świątkowska-Stodulska et al., 2018). Management for these conditions has been primarily aimed at symptoms that occur late in the course of the disorder. Women who develop preeclampsia during pregnancy are also at increased risk of eclampsia and cardiovascular events, both in the short and long term (Fishel Bartal and Sibai, 2020). Gestational diabetes prevalence rises with increased pregnancy age, maternal obesity and inactivity (Farrar, 2016). Also, there is a lack of consensus between healthcare professionals on methods of screening for gestational diabetes mellitus (Sert and Ozgu-Erdinc, 2021). Present research efforts are primarily focused on the advancement of early detection and therapeutic strategies. Identification of accurate biochemical markers for predicting and early diagnosing complications of pregnancy may have a major effect on maternal and fetal health.

Several lines of evidence have shown the interaction and role of the renin-angiotensin and melatonin systems in the pathways of several cardiovascular pathologies (Campos et al., 2013). Scientific evidence indicates the involvement of renin-angiotensin-aldosterone in pregnancy-induced hypertension and diabetes etiopathogenesis (Świątkowska-Stodulska et al., 2018). Urinary angiotensinogen, AT1 receptor antibodies, plasma renin and prorenin, maternal circulatory angiotensin II, and gene polymorphisms have been implicated (Gathiram and Moodley, 2020). Urinary angiotensinogen, a precursor to the renin-angiotensin system, has been identified by us and others as a potential early marker of diabetic nephropathy and chronic kidney disease (Kobori and Navar, 2011; de Alencar Franco Costa et al., 2015). A recent cross-sectional study showed that urinary angiotensinogen/creatinine ratio was correlated with high blood pressure and proteinuria in preeclampsia (Yilmaz et al., 2015). The renin-angiotensin system has also been implicated in the pathogenesis of preeclampsia (Verdonk et al., 2014; Blois et al., 2015). However, the results of investigations on the significance of the renin-angiotensin system in gestational diabetes and preeclampsia have been contentious (Gathiram and Moodley, 2020).

Melatonin, a circadian pineal hormone with free radical scavenging and antioxidant effects, has been investigated extensively in patients with cardiovascular disease and kidney injury (Baltatu et al., 2017). Emerging evidence supports melatonin in the pathophysiology of pregnancy and fetal development (Tain et al., 2017). Melatonin was found to be associated with maternal plasma antioxidant status in the first trimester of pregnancy and to be deficient in the development of obstetric complications (Ramiro-Cortijo et al., 2016). It has also been proposed that the decrease of circulating melatonin levels could be correlated with the development of preeclampsia. (Zeng et al., 2016). Significant effects of melatonin in placenta have been demonstrated in pre-eclampsia physiopathology (Sagrillo-Fagundes et al., 2014). A systematic review indicated a relationship between melatonin receptor 1B and insulin receptor substrate 1 polymorphisms associated with an increased risk of developing gestational diabetes mellitus (Zhang et al., 2014). Newly, melatonin synthesis impairment is considered as a deleterious outcome of diabetes-derived hyperglycemia (Campos et al., 2013; Amaral et al., 2014).

In addition to proteinuria, which is the most prevalent test during pregnancy (Fishel Bartal et al., 2020), novel urine biomarkers for early diagnosis of disorders that complicate pregnancy are being proposed (Aitekenov et al., 2021). Based on the evidence presented, we here hypothesized that alterations in urinary melatonin or angiotensinogen can occur in early stages of gestational diabetes or hypertension and that these biomarkers may potentially be helpful in the prediction and early identification of patients with both preeclampsia and gestational diabetes. Therefore, the primary objective of our study was to assess melatonin and angiotensinogen as potential novel biomarkers of disease that are present and quantifiable in the urine of pregnant women. In addition, we investigated the potential synergy of combined biomarkers strategy for pregnancy complications.

## Methods

**FIGURE 1:**
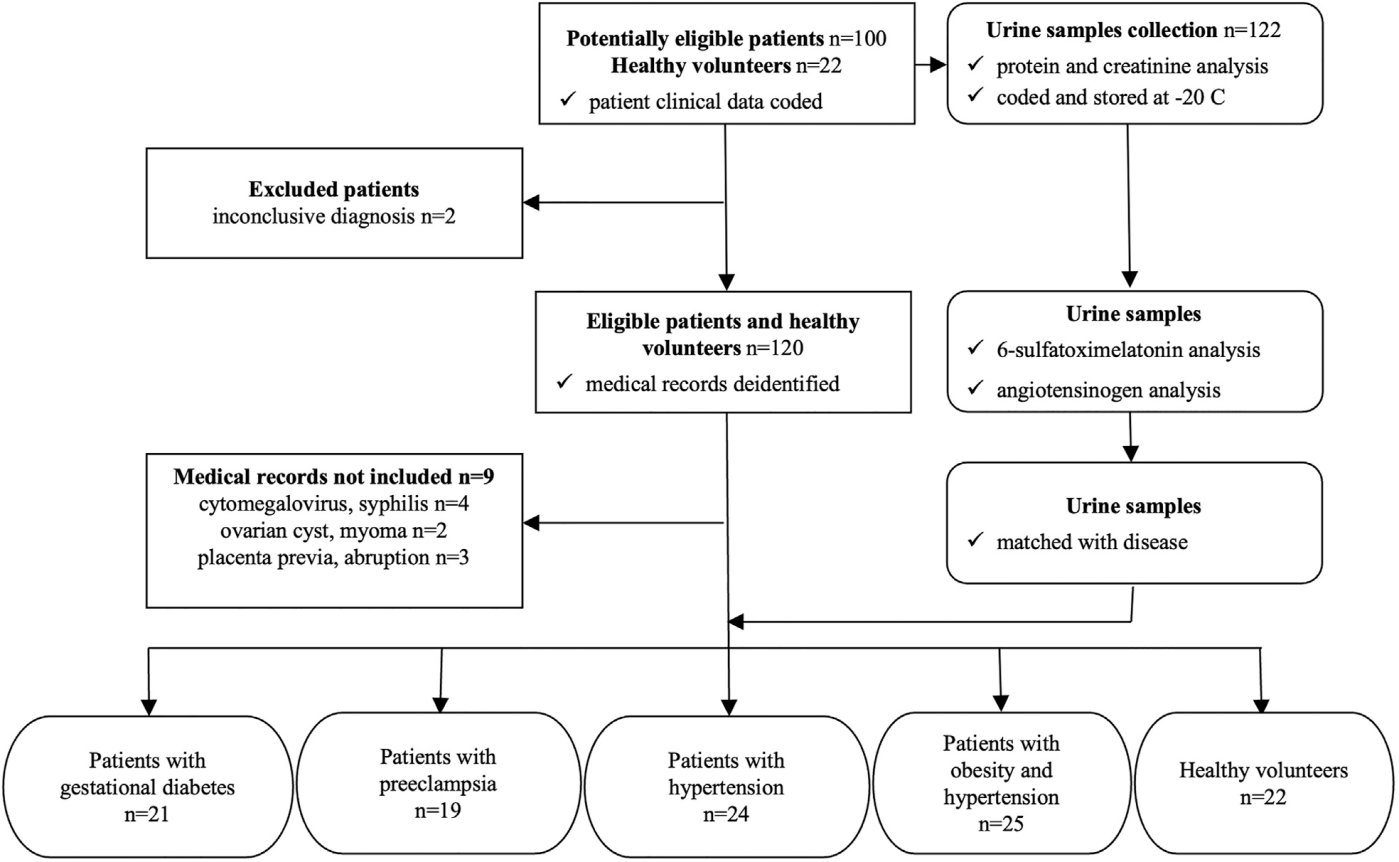
STARD diagram to report flow of participants through the study.

This is a concurrent cohort study using consecutive sampling. All patients were recruited from the prenatal care system of a single institution and included in the cohort based on inclusion criteria and consent to participate in the study. The subjects included were then followed up for the development of pregnancy complications (Figure 1). A subset of the cohort corresponded to normal patients. Biomarkers were assessed and their levels studied for association to the final presence or absence of disease complications in the pregnancies. The study sample size was estimated based on a prior study in which the standard deviation of the main variable (urinary angiotensinogen/creatinine ratio) was 2.1, detecting significant differences between three groups of study participants totaling 90 (Yilmaz et al., 2015). The probability is 80 percent that the study will detect a difference between groups of study at a two-sided 0.05 significance level, if the true difference between treatments is 1.2 units. The calculations led to a total of 100 patients to be recruited in this study. Considering 10% dropouts due to various factors, we calculated to recruit approximately 110 pregnant women in the study. Also, since this is a cohort observational model of the study, we expected that we would recruit in 1 year at least 110 pregnant women, as estimated by Hospital São Francisco de Assis - Jacareí.

The study was approved by the Ethical Committees of the Anhembi Morumbi University (CAAE 79831417.7.0000.5492), University of Sao Paulo (CAAE 30460114.5.0000.0068) and Hospital São Francisco de Assis. It was conducted in accordance with International Council for Harmonization of Technical Requirements for Pharmaceuticals for Human Use Guidelines and the Declaration of Helsinki. All patients provided written informed consent before entry into the study.

### Patient Selection

Pregnant women attending prenatal care at Sao Francisco de Assis Hospital, Jacareí/Sao Paulo were invited to participate to the study (During ---period of time of the recruitment--). Their selection was consecutive at the time of admission, and the diagnosis was blind to the level of urine collection and testing for the biomarkers. One hundred and 22 potentially eligible women were recruited that included 100 patients with gestational diabetes and/or preeclampsia, and 22 pregnant women without complications. Although this was not a diagnostic accuracy study, a STARD diagram was modified to display the flow of participants in the study (Figure 1) (Bossuyt et al., 2015). From the 122 study participants, first morning void urine samples were collected for protein and creatinine measurements, after which were aliquoted in duplicates, coded and stored at -20°C. The clinical diagnosis of study participants were coded (potentially eligible patients) and deidentified (eligible patients and healthy volunteers) according to the Brazilian General Data Protection Law (Galvin and DeMuro, 2020). The urine samples were encoded only by numbers and the link between numbers and patient names stored in a password protected computer program, according to the Brazilian General Data Protection Law (Galvin and DeMuro, 2020). After the urinary 6-sulfatoxymelatonin and angiotensinogen measurements, data were matched with the diagnosed diseases of 111 study participants (Figure 1). The characteristics of the study cohort was as follows: ages eligible for study—18 years and older, accepts healthy volunteers–yes, sampling method–non-probability sample. The following criteria of inclusion and exclusion were applied for the selection and enrollment of pregnant women in the study. Inclusion criteria: gestational age between 6 and 22 weeks for the first visit determined by ultrasound, high risk group (presence of specific risk factors for preterm delivery, pregnancy-induced hypertension or intrauterine growth retardation), low risk group (normal pregnancy with no risk factors for preterm delivery, pregnancy-induced hypertension or intrauterine growth retardation). Exclusion criteria were: known malignancy, pregnancies without ultrasonographic confirmation of gestational age, multifetal pregnancy with greater than or equal to 3 fetuses, active vaginal bleeding, serious medical illness (renal insufficiency, congestive heart disease, chronic respiratory insufficiency), asthma requiring systemic steroids, patient requiring anti-platelet or non-steroidal anti-inflammatory drugs, active hepatitis, and lack of consent.

### Clinical Evaluations

The diagnosis of preeclampsia or gestational diabetes were made by the hospital’s obstetricians using the following criteria. Preeclampsia was defined as *de novo* hypertension with high blood pressure (systolic ≥140 or diastolic ≥90 mm Hg) on two occasions, at least 4 hours apart, after 20 weeks of pregnancy and proteinuria of 0.3 g in 24 h urine collection, or 2+ in 2 samples with 2 h apart, or more than 0.3 mg in a single sample (Sousa et al., 2019; Leal et al., 2020). Gestational diabetes mellitus was defined as carbohydrate intolerance, resulting in hyperglycemia of varying severity with onset or first recognition during pregnancy. Development of gestational diabetes at 24–28 weeks of gestation for abnormal values in the 75 g oral glucose tolerance test (American Diabetes Association, 2013), as recommended by the Brazilian Ministry of Health (Dos Santos et al., 2020). Patients with preexistent hypertension were those diagnosed with hypertension prior or up to the 20th week of pregnancy (Sousa et al., 2019). Obesity was defined by body mass index (BMI) values ≥30 kg/m^2^ and identified as a condition prior to pregnancy (Ferreira et al., 2019; Indarti et al., 2021). First morning void urine samples were collected for the study at the time of admission after signing the consent form.

### Clinical Laboratory, 6-Sulfatoxymelatonin and Angiotensinogen Measurements

Urine samples were obtained in the event of pregnancy complications, at which time pregnant women sought medical help; urine was collected as the first morning urine, and was used for the protein, creatinine, 6-sulfatoxymelatonin and angiotensinogen urinary tests (Figure 1).

Proteinuria (mg/dl), urinary creatinine (mg/dl) and the protein/creatinine ratio were determined by the hospital’s laboratory. From these measures, the mg/24-h ratio of creatinine excretion and the 24-h urinary protein estimate (mg/24 h) were determined using the Fotheringham’s et al. equation (Fotheringham et al., 2014).

Urinary 6-sulfatoxymelatonin and angiotensinogen were measured by sensitive and specific ELISAs in first morning void urine samples. All maternal samples were analyzed in duplicate, and laboratory personnel were unaware of the diagnostic status of the study subjects at the time of analysis. Urinary excretion of angiotensinogen was analyzed by sandwich-ELISA assay (Kobori et al., 2009; de Alencar Franco Costa et al., 2015). Melatonin excreted overnight was determined through urinary 6-sulfatoxymelatonin levels (Baltatu et al., 2002; Campos et al., 2020). The ELISA kits for both angiotensinogen and 6-sulfatoxymelatonin were from IBL International GmbH Germany.

### Statistical Analysis

Data were tested for normality distribution using D'Agostino-Pearson normality test (D’agostino, 2017) and Kolmogorov-Smirnov test with Dallal-Wilkinson-Lilliefors’ *p* value (Dallal and Wilkinson, 1986). Differences between study groups, were assessed with Kruskal–Wallis nonparametric test, followed by a comparison of the mean rank of each group with the mean rank of the control group (healthy pregnant subjects). Multiple testing correction was done by controlling the False Discovery Rate with a two-stage step-up method of Benjamini, Krieger and Yekutieli (Benjamini et al., 2006). All statistical analyses were carried out using GraphPad Prism version 8.1.2 for Mac OS X, GraphPad Software, La Jolla California United States, www.graphpad.com. Differences were considered significant when the probability of a Type I error was lower than 5% (*p* < 0.05).

## Results

### Patient Demographics and Laboratory Data

The recruited study cohort included patients with gestational diabetes mellitus (%,n) (17.2%, 21), with preeclampsia (15.6%, 19), with preexistent hypertension (19.7%, 24), with preexistent obesity and hypertension (20.5%, 25), and healthy volunteers (18.0%, 22).

**FIGURE 2:**
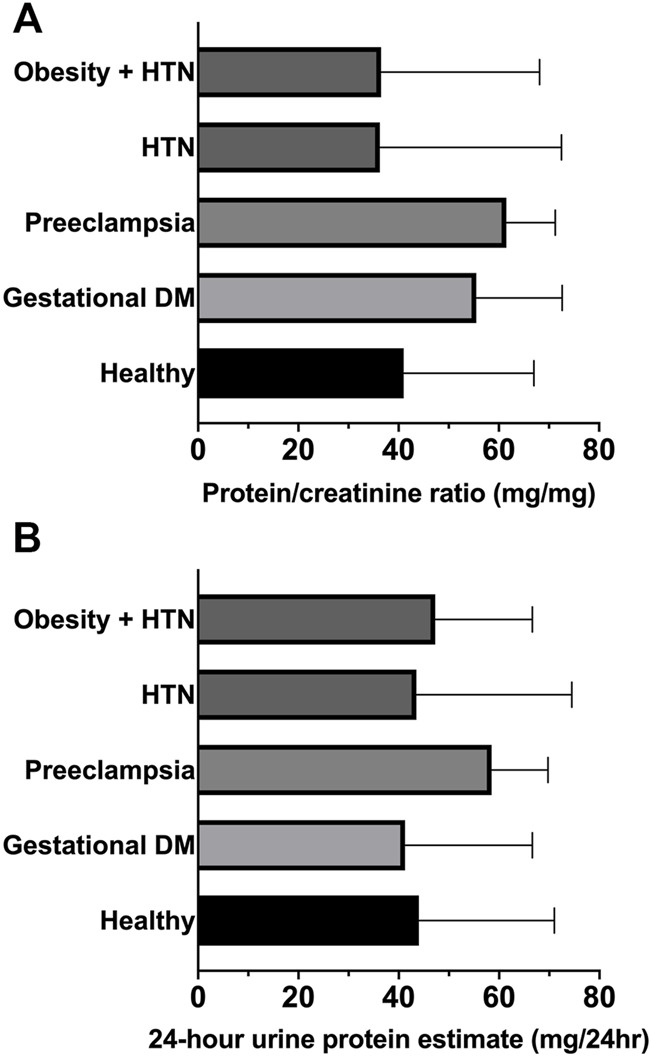
**(A)** Protein/creatinine ratio. **(B)** 24-h urine protein estimate. Graphs are ranks plots with bars of median with interquartile range; Kruskal–Wallis test, no significant differences between groups.

Table 1 indicates for each study group the age, gestational age, multiparity, previous cesarean delivery or abortion. No statistical differences were observed between the study groups for age or gestational age. The patients’ gestational age was: 24–28 weeks for patients with gestational diabetes mellitus, 20–33 weeks for patients with preeclampsia, and 20–33 weeks for patients with hypertension, with obesity plus hypertension, or healthy volunteers. Multiparity, previous cesarean delivery, or abortion appear to be more prevalent in disease groups than in healthy group. Urine volume (ml), creatinine (mg/dl), protein/creatinine ratio (mg/mg) and 24-h urine protein estimate are presented as median and interquartile range (IQR, 25th-75th percentile) per group of study. In gestational diabetes, urine volume was significantly greater and urine creatinine was significantly lower than in healthy control pregnancies. Protein-creatinine ratio was not different between the study groups–Table 1 and Figure 2A. The 24-h urine protein estimate (mg/24h) was not different between the study groups–Table 1 and Figure 2B.

**TABLE 1:** Patient demographics and laboratory urine analyses in the studied cohort.

Parameter median (25th, 75th)	Healthy	Gestational diabetes	Preeclampsia	Hypertension	Hypertension + obesity (n = 25)
	(n = 22)	(n = 21)	(n = 19)	(n = 24)	
Age (years)	28.00 (24.00, 31.50)	32.00 (27.50, 35.00)	29.00 (26.00, 33.00)	32.50 (28.50, 36.75)	32.00 (28.50, 37.00)
Gestational age (weeks)	27.50 (23.75, 32.00)	25.00 (24.00, 26.50)	29.00 (26.00, 33.00)	30.50 (28.00, 33.00)	31 (28.50, 33.00)
Multiparous [N (%)]	9 (40.9%)	20 (95.2%)	19 (100%)	21 (87.5%)	16 (64%)
Previous cesarean delivery [N (%)]	3 (13.6%)	10 (47.6%)	10 (52.6%)	11 (45.8%)	11 (44%)
Previous abortion [N (%)]	2 (9.1%)	9 (42.9%)	10 (52.6%)	6 (25%)	5 (20%)
Urine angiotensinogen/creatinine ratio (μg/g)	0.05 (0.04, 0.06)	0.11 (0.07, 0.18) **	0.08 (0.06, 0.18)*	0.05 (0.04, 0.06)	0.06 (0.05, 0.09)
Urine 6-sulfatoximelatonin (μg/h)	0.20 (0.15, 0.27)	0.12 (0.08, 0.17)*	0.12 (0.09, 0.15)*	0.19 (0.15, 0.32)	0.21 (0.17, 0.30)
Urine angiotensinogen/6-sulfatoximelatonin/creatinine Ratio	0.27 (0.16, 0.32)	0.94 (0.36, 2.06) **	0.72 (0.50, 1.40) **	0.29 (0.15, 0.39)	0.31 (0.20, 0.48)
Urine volume (ml)	50.00 (40.00, 62.50)	70.00 (55.00, 70.00)	50.00 (40.00, 60.00)	57.50 (41.25, 70.00)	50.00 (40.00, 60.00)
Urine creatinine (mg/dl)	93.75 (70.63, 133.7)	57.00 (34.00, 82.88)*	80.50 (34.00, 116.5)	95.63 (59.88, 127.4)	123.5 (76.5, 150.0)
Protein/creatinine Ratio (mg/mg)	0.08 (0.05, 0.15)	0.12 (0.08, 0.19)	0.14 (0.05, 0.19)	0.10 (0.06, 0.18)	0.09 (0.04, 0.12)
24-h urine protein estimate	145.8 (121.1, 246.2)	129.0 (68.52, 187.4)	155.5 (129.0, 216.5)	128.5 (91.87, 224.2)	133.0 (61.81, 174.7)

*p < 0.05, **p < 0.005.

### Urine 6-Sulfatoxymelatonin and Angiotensinogen

**FIGURE 3:**
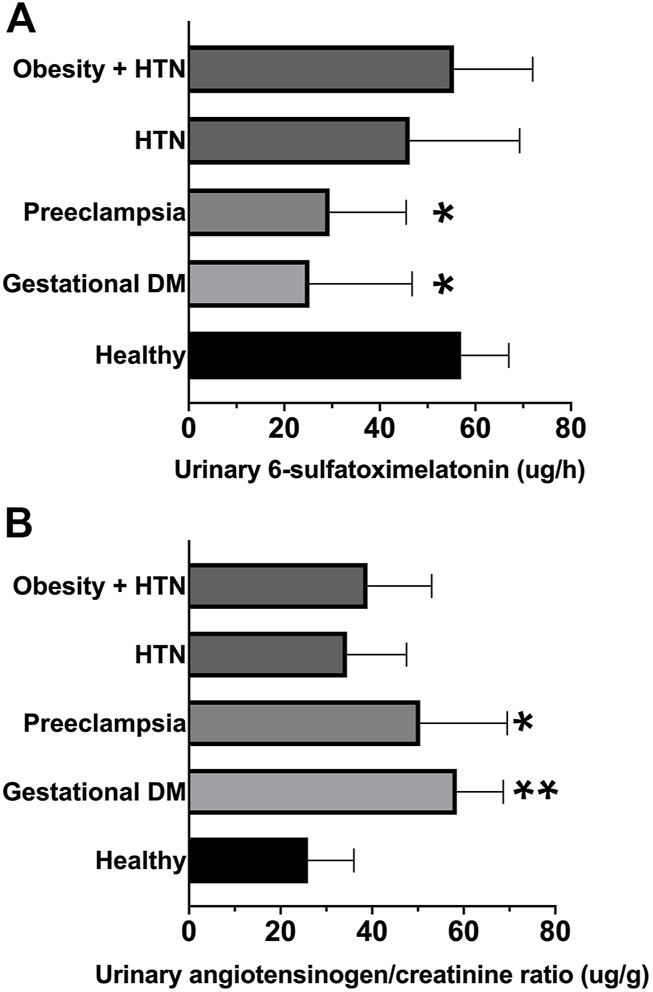
**(A)** Urinary 6-sulfatoxymelatonin (ug/h) in first morning void urine samples. **(B)** Urinary angiotensinogen-creatinine ratio (ng/mg). Graphs are ranks plots with bars of median with interquartile range; Kruskal–Wallis test *, *p* < 0.05 and **, *p* < 0.005 in comparison to the healthy group.

Urine 6-sulfatoxymelatonin (ug/h) was significantly reduced in the first morning void urine samples of patients with gestational diabetes and preeclampsia–Figure 3A and Table 1. Urinary angiotensinogen (angiotensinogen/creatinine ratio, ug/g) was significantly increased in the first morning void urine samples of patients with gestational diabetes and preeclampsia–Figure 3B and Table 1.

**FIGURE 4:**
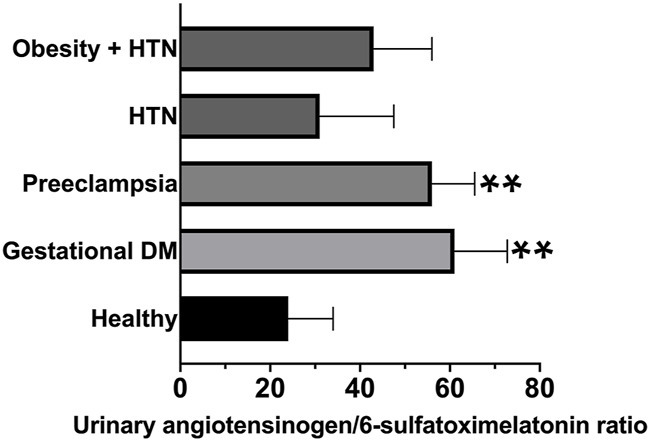
Angiotensinogen/6-sulfatoxymelatonin ratio was calculated as (angiotensinogen/creatinine ratio, ug/g)/(6-sulfatoxymelatonin, ug/h). Graphs are ranks plots with bars of median with interquartile range; Kruskal–Wallis test **, *p* < 0.005 in comparison to the healthy group.

The ratio of angiotensinogen/6-sulfatoxymelatonin was calculated as (angiotensinogen/creatinine ratio, ug/g)/(6-sulfatoxymelatonin, ug/h) significantly increased in the first morning void urine samples of patients with gestational diabetes and preeclampsia–Figure 4 and Table 1.

## Discussion

The main findings of this study are the high angiotensinogen and low melatonin levels in the urine of women with gestational diabetes or preeclampsia. These findings may support the development of diagnostic tools and strategies for recognizing patients at risk of pregnancy complications (Phipps et al., 2016).

Renin-angiotensin system has been involved in the development of preeclampsia. Studies have shown that angiotensinogen, the precursor of all angiotensin peptides (Lu et al., 2016), is a particularly good candidate biomarker for both its acute (Ba Aqeel et al., 2017) and chronic (Juretzko et al., 2017) kidney injury (Chen et al., 2016). Research indicates that urinary angiotensinogen comes mainly from the kidney, and higher levels correlate with diabetic albuminuria (Kobori and Navar, 2011; de Alencar Franco Costa et al., 2015). The current study’s findings of an elevated urine angiotensinogen/creatinine ratio in preeclampsia corroborate the findings of (Yilmaz et al. (2015) and of Mistry et al. (2019). These findings contrast with those of other research, such as Chen et al. (2011)) or Zhang et al. (2017), which found considerably reduced urine angiotensinogen levels in preeclampsia. Pringle et al. found a rise in urine angiotensinogen/creatinine levels as pregnancy progressed, but a significant decline in the third trimester in a group of women with pregnancy complications that included preeclampsia and gestational diabetes (Pringle et al., 2018). These may imply a time-dependent activation/suppression of the renal renin-angiotensin system during preeclampsia, as measured by urine angiotensinogen/creatinine levels. It appears that additional research is necessary to determine the relevance of urinary angiotensinogen in the detection of preeclampsia. Although Pringle et al. included women with gestational diabetes in their investigation, no pathology-specific group analyses were performed on urine angiotensinogen/creatinine levels. As a result, the current study may be the first to report significantly elevated urine angiotensinogen/creatinine levels in gestational diabetes.

A large body of evidence suggests that melatonin has a protective effect during the development of kidney disease, at least in part by counteracting the effects of intrarenal renin-angiotensin system activation, prompting the need for further research into the roles of melatonin and angiotensinogen in renal injury (Campos et al., 2013; Baltatu et al., 2017; Ohashi et al., 2019). Its role in pregnancy conditions is still to be clearly understood (Tong et al., 2020). The current study’s findings of low levels in preeclampsia corroborate Bouchlariotou’s et al. findings (Bouchlariotou et al., 2014). Additionally, Bouchlariotou et al. revealed a link between impaired melatonin secretion and an non-dipper blood pressure (no nocturnal physiological blood pressure decrease) in women with preeclampsia. Our study is the first to demonstrate that women with gestational diabetes had decreased urinary 6-sulfatoxymelatonin levels. While the findings of Bouchlariotou et al. and ours may not be sufficient to implicate melatonin in the pathogenesis of preeclampsia, they certainly suggest that its participation in the pathogenesis of preeclampsia and gestational diabetes warrants further investigation.

The presence of elevated angiotensinogen and low 6-sulfatoxymelatonin in the first morning void urine samples of patients with gestational diabetes or preeclampsia suggests activation of the intrarenal RAS and an impairment in melatonin synthesis. Additionally, the urine angiotensinogen-6-sulfatoxymelatonin ratio was elevated in women with gestational diabetes or preeclampsia. Although urine protein/creatinine levels increased in the gestational diabetes and preeclampsia groups as well, the difference was not statistically significant when compared to the control group. This could be because the study groups included a limited number of cases. This apparent disparity between proteinuria results from the first morning void and physician diagnosis of preeclampsia could be related to sampling methods, as well. In fact, Lamontagne et al. demonstrated that early morning urine protein was ineffective in detecting preeclampsia (Lamontagne et al., 2014). Although several studies on “spot urine” protein to creatinine ratio estimation suggest a promising diagnostic value for preeclampsia (Stefańska et al., 2020), the test’s heterogeneity in accuracy precludes yet its utility as a diagnostic tool (Morris et al., 2012).

There are important limitations of our study that should be recognized. Since our study is cross-sectional, assigning causality between the altered urinary angiotensinogen or melatonin levels and pathogenesis of gestational diabetes and preeclampsia is not possible. Although the recruited cohort might be considered a representative sample for the state of Sao Paulo, this single-center study requires confirmation in various cohorts or multicenter investigations. Although the total number of patients is not insignificant, subgroups of individuals include a limited number of cases, thus the results should be considered as a pilot study requiring further analysis and confirmation. Using multivariable adjusted models, we will be able to assess the impact of other factors on the reported correlations between altered urine angiotensinogen or melatonin levels and gestational diabetes and preeclampsia.

Further longitudinal diagnostic cohort studies are expected to define the operative characteristics of these markers for risk assessment, prediction and diagnosis of the studied conditions. Also, cohort studies may allow to track the pathological evolution of the disease and to explore its potential association with urinary melatonin or angiotensinogen. Further studies need to investigate as well the optimal cutoff value to use urinary angiotensinogen/melatonin ratio as clinical decision guidance.

## Conclusion

These findings suggest that the levels of urinary angiotensinogen may indicate intra-renal RAS activation, whereas the production of melatonin appears to be defective in gestational diabetes or preeclampsia. For early diagnosis of gestational diabetes or preeclampsia, morning void urine angiotensinogen-melatonin-creatinine ratio is suggested. This report provides a rationale for the possible use of melatonin as a potential coadjuvant therapeutic agent for the treatment of preeclampsia or gestational diabetes.

## Data Availability

The datasets generated during and/or analyzed during the current study are available from the corresponding author on reasonable request.

## References

[B1] Aitekenov S. Gaipov A. Bukasov R. (2021). Review: Detection and Quantification of Proteins in Human Urine. Talanta 223, 121718. 10.1016/j.talanta.2020.121718 33303164 PMC7554478

[B2] Amaral F. G. Turati A. O. Barone M. Scialfa J. H. do Carmo Buonfiglio D. Peres R. (2014). Melatonin Synthesis Impairment as a New Deleterious Outcome of Diabetes-Derived Hyperglycemia. J. Pineal Res. 57, 67–79. 10.1111/jpi.12144 24819547

[B3] American Diabetes Association (2013). Diagnosis and Classification of Diabetes Mellitus. Diabetes Care 36 Suppl 1, S67–S74. 10.2337/dc13-S067 23264425 PMC3537273

[B4] Ba Aqeel S. H. Batlle D. Batlle D. (2017). Angiotensinogen as a Biomarker of Acute Kidney Injury. Clin. Kidney J. 10, 759–768. 10.1093/ckj/sfx087 29225804 PMC5716162

[B5] Baltatu O. Afeche S. C. Santos S. H. J. d. Campos L. A. Barbosa R. Michelini L. C. (2002). Locally Synthesized Angiotensin Modulates Pineal Melatonin Generation. J. Neurochem. 80, 328–334. 10.1046/j.0022-3042.2001.00701.x 11902123

[B6] Baltatu O. C. Amaral F. G. Campos L. A. Cipolla-Neto J. (2017). Melatonin, Mitochondria and Hypertension. Cell. Mol. Life Sci. 74, 3955–3964. 10.1007/s00018-017-2613-y 28791422 PMC11107636

[B7] Benjamini Y. Krieger A. M. Yekutieli D. (2006). Adaptive Linear Step-Up Procedures that Control the False Discovery Rate. Biometrika 93, 491–507. 10.1093/biomet/93.3.491

[B8] Blois S. M. Dechend R. Barrientos G. Staff A. C. (2015). A Potential Pathophysiological Role for Galectins and the Renin-Angiotensin System in Preeclampsia. Cel. Mol. Life Sci. 72, 39–50. 10.1007/s00018-014-1713-1 PMC11113509 25192660

[B9] Bossuyt P. M. Reitsma J. B. Bruns D. E. Gatsonis C. A. Glasziou P. P. Irwig L. (2015). STARD 2015: an Updated List of Essential Items for Reporting Diagnostic Accuracy Studies. BMJ 351, h5527. 10.1136/bmj.h5527 26511519 PMC4623764

[B10] Bouchlariotou S. Liakopoulos V. Giannopoulou M. Arampatzis S. Eleftheriadis T. Mertens P. R. (2014). Melatonin Secretion Is Impaired in Women with Preeclampsia and an Abnormal Circadian Blood Pressure Rhythm. Ren. Fail. 36, 1001–1007. 10.3109/0886022X.2014.926216 24932757

[B11] Campos L. A. Bueno C. Barcelos I. P. Halpern B. Brito L. C. Amaral F. G. (2020). Melatonin Therapy Improves Cardiac Autonomic Modulation in Pinealectomized Patients. Front. Endocrinol. 11, 239. 10.3389/fendo.2020.00239 PMC7213221 32431667

[B12] Campos L. A. Cipolla-Neto J. Amaral F. G. Michelini L. C. Bader M. Baltatu O. C. (2013). The Angiotensin-Melatonin axis. Int. J. Hypertens. 2013, 1–7. 10.1155/2013/521783 PMC3556444 23365722

[B13] Chen C. Yang X. Lei Y. Zha Y. Liu H. Ma C. (2016). Urinary Biomarkers at the Time of AKI Diagnosis as Predictors of Progression of AKI Among Patients with Acute Cardiorenal Syndrome. Cjasn 11, 1536–1544. 10.2215/CJN.00910116 27538426 PMC5012473

[B14] Chen G. Zhang Y. Jin X. Zhang L. Zhou Y. Niu J. (2011). Urinary Proteomics Analysis for Renal Injury in Hypertensive Disorders of Pregnancy with iTRAQ Labeling and LC-MS/MS. Prot. Clin. Appl. 5, 300–310. 10.1002/prca.201000100 21538910

[B15] D’agostino R. (2017). Tests for the normal Distribution. Goodness, 367–420. 10.1201/9780203753064-9

[B16] Dallal G. E. Wilkinson L. (1986). An Analytic Approximation to the Distribution of Lilliefors’s Test Statistic for Normality. Am. Stat. 10.2307/2684607

[B17] de Alencar Franco Costa D. Todiras M. Campos L. A. Cipolla-Neto J. Bader M. Baltatu O. C. (2015). Sex-dependent Differences in Renal Angiotensinogen as an Early Marker of Diabetic Nephropathy. Acta Physiol. (Oxf) 213, 740–746. 10.1111/apha.12441 25529203

[B18] dos Santos P. A. da Silva E. R. Vergani D. d. O. P. Garcia R. M. R. de Araújo B. F. (2020). Gestational Diabetes in the Population Served by Brazilian Public Health Care. Prevalence and Risk Factors. Rev. Bras. Ginecol. Obstet. 42, 012–018. 10.1055/s-0039-1700797 PMC10316875 32107761

[B19] Farrar D. (2016). Hyperglycemia in Pregnancy: Prevalence, Impact, and Management Challenges. Ijwh Vol. 8, 519–527. 10.2147/IJWH.S102117 PMC5036767 27703397

[B20] Ferreira A. P. d. S. Szwarcwald C. L. Damacena G. N. Damacena G. N. (2019). Prevalência e fatores associados da obesidade na população brasileira: estudo com dados aferidos da Pesquisa Nacional de Saúde, 2013. Rev. Bras. Epidemiol. 22, e190024. 10.1590/1980-549720190024 30942330

[B21] Fishel Bartal M. Lindheimer M. D. Sibai B. M. (2020). Proteinuria During Pregnancy: Definition, Pathophysiology, Methodology, and Clinical Significance. Am. J. Obstet. Gynecol. S0002-9378 (20), S0002-9378(20)30989-3. 10.1016/j.ajog.2020.08.108 32882208

[B22] Fishel Bartal M. Sibai B. M. (2020). Eclampsia in the 21st century. Am. J. Obstet. Gynecol., S0002-9378(20)31128-5. 10.1016/j.ajog.2020.09.037 32980358

[B23] Fotheringham J. Campbell M. J. Fogarty D. G. El Nahas M. Ellam T. (2014). Estimated Albumin Excretion Rate versus Urine Albumin-Creatinine Ratio for the Estimation of Measured Albumin Excretion Rate: Derivation and Validation of an Estimated Albumin Excretion Rate Equation. Am. J. Kidney Dis. 63, 405–414. 10.1053/j.ajkd.2013.08.009 24084157

[B24] Galvin H. K. DeMuro P. R. (2020). Developments in Privacy and Data Ownership in Mobile Health Technologies, 2016-2019. Yearb. Med. Inform. 29, 032–043. 10.1055/s-0040-1701987 PMC7442507 32823298

[B25] Gathiram P. Moodley J. (2020). The Role of the Renin-Angiotensin-Aldosterone System in Preeclampsia: a Review. Curr. Hypertens. Rep. 22, 89. 10.1007/s11906-020-01098-2 32893333

[B26] Indarti J. Susilo S. A. Hyawicaksono P. Berguna J. S. N. Tyagitha G. A. Ikhsan M. (2021). Maternal and Perinatal Outcome of Maternal Obesity at RSCM in 2014-2019. Obstet. Gynecol. Int. 2021, 1–6. 10.1155/2021/6039565 PMC7886500 33628260

[B27] Juretzko A. Steinbach A. Hannemann A. Endlich K. Endlich N. Friedrich N. (2017). Urinary Angiotensinogen and Renin Excretion Are Associated with Chronic Kidney Disease. Kidney Blood Press. Res. 42, 145–155. 10.1159/000474932 28395289

[B28] Kobori H. Navar L. G. (2011). Urinary Angiotensinogen as a Novel Biomarker of Intrarenal Renin-Angiotensin System in Chronic Kidney Disease. Int. Rev. Thromb. 6 (2), 108–116. 22022346 PMC3183743

[B29] Kobori H. Alper A. B. Shenava R. Katsurada A. Saito T. Ohashi N. (2009). Urinary Angiotensinogen as a Novel Biomarker of the Intrarenal Renin-Angiotensin System Status in Hypertensive Patients. Hypertension 53, 344–350. 10.1161/HYPERTENSIONAHA.108.123802 19075095 PMC2658771

[B30] Lamontagne A. Côté A.-M. Rey E. (2014). The Urinary Protein-To-Creatinine Ratio in Canadian Women at Risk of Preeclampsia: Does the Time of Day of Testing Matter? J. Obstet. Gynaecol. Can. 36, 303–308. 10.1016/S1701-2163(15)30605-8 24798667

[B31] Leal L. F. Grandi S. M. Miranda V. I. A. Dal Pizzol T. d. S. Platt R. W. Silveira M. F. d. (2020). Hypertensive Disorders of Pregnancy and Medication Use in the 2015 Pelotas (brazil) Birth Cohort Study. Ijerph 17, 8541. 10.3390/ijerph17228541 PMC7698775 33217917

[B32] Lu H. Cassis L. A. Kooi C. W. V. Daugherty A. (2016). Structure and Functions of Angiotensinogen. Hypertens. Res. 39, 492–500. 10.1038/hr.2016.17 26888118 PMC4935807

[B33] Mistry H. D. Kurlak L. O. Gardner D. S. Torffvit O. Hansen A. Broughton Pipkin F. (2019). Evidence of Augmented Intrarenal Angiotensinogen Associated with Glomerular Swelling in Gestational Hypertension and Preeclampsia: Clinical Implications. Jaha 8, e012611. 10.1161/JAHA.119.012611 31237175 PMC6662362

[B34] Morris R. K. Riley R. D. Doug M. Deeks J. J. Kilby M. D. (2012). Diagnostic Accuracy of Spot Urinary Protein and Albumin to Creatinine Ratios for Detection of Significant Proteinuria or Adverse Pregnancy Outcome in Patients with Suspected Pre-eclampsia: Systematic Review and Meta-Analysis. BMJ 345, e4342. 10.1136/bmj.e4342 22777026 PMC3392077

[B35] Ohashi N. Ishigaki S. Isobe S. (2019). The Pivotal Role of Melatonin in Ameliorating Chronic Kidney Disease by Suppression of the Renin-Angiotensin System in the Kidney. Hypertens. Res. 42, 761–768. 10.1038/s41440-018-0186-2 30610209

[B36] Phipps E. Prasanna D. Brima W. Jim B. (2016). Preeclampsia: Updates in Pathogenesis, Definitions, and Guidelines. Cjasn 11, 1102–1113. 10.2215/CJN.12081115 27094609 PMC4891761

[B37] Pringle K. G. de Meaultsart C. C. Weatherall L. J. Keogh L. Clausen D. C. (2018). Urinary Angiotensinogen Excretion in Australian Indigenous and Non-indigenous Pregnant Women. Pregnancy Hypertens. 12, 110–117. 10.1016/j.preghy.2018.04.009 29674190

[B38] Ramiro-Cortijo D. Herrera T. Rodríguez-Rodríguez P. López De Pablo Á. L. López-Giménez M. R. Mora-Urda A. I. (2016). Maternal Plasma Antioxidant Status in the First Trimester of Pregnancy and Development of Obstetric Complications. Placenta 47, 37–45. 10.1016/j.placenta.2016.08.090 27780538

[B39] Sagrillo-Fagundes L. Soliman A. Vaillancourt C. (2014). Maternal and Placental Melatonin: Actions and Implication for Successful Pregnancies. Minerva Ginecol 66, 251–266. 24971781

[B40] Sert U. Y. Ozgu-Erdinc A. S. (2021). Gestational Diabetes Mellitus Screening and Diagnosis. Adv. Exp. Med. Biol. 1307, 231–255. 10.1007/5584_2020_512 32314318

[B41] Sousa M. G. de, Lopes R. G. C. Rocha M. L. T. L. F. da, Lippi U. G. Costa E. de S. (2019). Epidemiology of Artherial Hypertension in Pregnants. Einstein (Sao Paulo) 18, eAO4682. 10.31744/einstein_journal/2020ao4682 31664330 PMC6896657

[B42] Stefańska K. Zieliński M. Zamkowska D. Adamski P. Jassem-Bobowicz J. Piekarska K. (2020). Comparisons of Dipstick Test, Urine Protein-To-Creatine Ratio, and Total Protein Measurement for the Diagnosis of Preeclampsia. Ijerph 17, 4195. 10.3390/ijerph17124195 PMC7344421 32545523

[B43] Świątkowska-Stodulska R. Kmieć P. Stefańska K. Sworczak K. (2018). Renin-Angiotensin-Aldosterone System in the Pathogenesis of Pregnancy-Induced Hypertension. Exp. Clin. Endocrinol. Diabetes 126, 362–366. 10.1055/s-0044-102008 29558785

[B44] Tain Y.-L. Huang L.-T. Hsu C.-N. (2017). Developmental Programming of Adult Disease: Reprogramming by Melatonin? Ijms 18, 426. 10.3390/ijms18020426 PMC5343960 28212315

[B45] Tong S. Kaitu’u-Lino T. J. Hastie R. Brownfoot F. Cluver C. Hannan N. (2020). Pravastatin, Proton-Pump Inhibitors, Metformin, Micronutrients, and Biologics: New Horizons for the Prevention or Treatment of Preeclampsia. Am. J. Obstet. Gynecol. 10.1016/j.ajog.2020.09.014. 10.1016/j.ajog.2020.09.014 32946849

[B46] Vadalà C. Cernaro V. Siligato R. Granese R. Laganà A. S. Buemi M. (2017). Rischio di Danno Renale a Distanze in Donne Con Preeclampsia [Long-Term Outcome of Renal Function in Women with Preeclampia and Pregestational Diabetes]. G Ital. Nefrol 2017-vol6. (Italian). 29207220

[B47] Verdonk K. Visser W. Van Den Meiracker A. H. Danser A. H. J. (2014). The Renin-Angiotensin-Aldosterone System in Pre-eclampsia: the Delicate Balance between Good and Bad. Clin. Sci. 126, 537–544. 10.1042/CS20130455 24400721

[B48] Yilmaz Z. Yildirim T. Yilmaz R. Aybal-Kutlugun A. Altun B. Kucukozkan T. (2015). Association between Urinary Angiotensinogen, Hypertension and Proteinuria in Pregnant Women with Preeclampsia. J. Renin Angiotensin Aldosterone Syst. 16, 514–520. 10.1177/1470320313510585 24532824

[B49] Zeng K. Gao Y. Wan J. Tong M. Lee A. C. Zhao M. (2016). The Reduction in Circulating Levels of Melatonin May Be Associated with the Development of Preeclampsia. J. Hum. Hypertens. 30, 666–671. 10.1038/jhh.2016.37 27251079

[B50] Zhang L. Zhou Y. Wu Q. Fan W. Ye J. Chen Y. (2017). Effective Prediction of Preeclampsia by Measuring Serum Angiotensin II, Urinary Angiotensinogen and Urinary Transforming Growth Factor β1. Exp. Ther. Med. 14, 391–397. 10.3892/etm.2017.4484 28672944 PMC5488619

[B51] Zhang Y. Sun C.-M. Hu X.-Q. Zhao Y. (2014). Relationship between Melatonin Receptor 1B and Insulin Receptor Substrate 1 Polymorphisms with Gestational Diabetes Mellitus: a Systematic Review and Meta-Analysis. Sci. Rep. 4, 6113. 10.1038/srep06113 25146448 PMC4141258

